# Antitumor Effect of Malaria Parasite Infection in a Murine Lewis Lung Cancer Model through Induction of Innate and Adaptive Immunity

**DOI:** 10.1371/journal.pone.0024407

**Published:** 2011-09-09

**Authors:** Lili Chen, Zhengxiang He, Li Qin, Qinyan Li, Xibao Shi, Siting Zhao, Ling Chen, Nanshan Zhong, Xiaoping Chen

**Affiliations:** 1 Center for Infection and Immunity, State Key Laboratory of Respiratory Disease, Guangzhou Institutes of Biomedicine and Health, Chinese Academy of Sciences, Guangzhou, China; 2 State Key Laboratory of Respiratory Disease, Guangzhou Institute of Respiratory Disease, First Affiliated Hospital, Guangzhou Medical University, Guangzhou, China; 3 CAS-FS Biotech and Pharmaceutical Center, Chinese Academy of Sciences, Foshan, China; National Jewish Health, United States of America

## Abstract

**Background:**

Lung cancer is the most common malignancy in humans and its high fatality means that no effective treatment is available. Developing new therapeutic strategies for lung cancer is urgently needed. Malaria has been reported to stimulate host immune responses, which are believed to be efficacious for combating some clinical cancers. This study is aimed to provide evidence that malaria parasite infection is therapeutic for lung cancer.

**Methodology/Principal Findings:**

Antitumor effect of malaria infection was examined in both subcutaneously and intravenously implanted murine Lewis lung cancer (LLC) model. The results showed that malaria infection inhibited LLC growth and metastasis and prolonged the survival of tumor-bearing mice. Histological analysis of tumors from mice infected with malaria revealed that angiogenesis was inhibited, which correlated with increased terminal deoxynucleotidyl transferase-mediated (TUNEL) staining and decreased Ki-67 expression in tumors. Through natural killer (NK) cell cytotoxicity activity, cytokine assays, enzyme-linked immunospot assay, lymphocyte proliferation, and flow cytometry, we demonstrated that malaria infection provided anti-tumor effects by inducing both a potent anti-tumor innate immune response, including the secretion of IFN-γ and TNF-α and the activation of NK cells as well as adaptive anti-tumor immunity with increasing tumor-specific T-cell proliferation and cytolytic activity of CD8^+^ T cells. Notably, tumor-bearing mice infected with the parasite developed long-lasting and effective tumor-specific immunity. Consequently, we found that malaria parasite infection could enhance the immune response of lung cancer DNA vaccine pcDNA3.1-hMUC1 and the combination produced a synergistic antitumor effect.

**Conclusions/Significance:**

Malaria infection significantly suppresses LLC growth via induction of innate and adaptive antitumor responses in a mouse model. These data suggest that the malaria parasite may provide a novel strategy or therapeutic vaccine vector for anti-lung cancer immune-based therapy.

## Introduction

Lung cancer is the leading cause of cancer-related deaths worldwide [1]. Although treatment methods in surgery, irradiation, and chemotherapy have been improved, prognosis remains unsatisfactory, and developing new therapeutic strategies is still an urgent demand. Immunotherapy may represent one of new therapeutic strategies for lung cancer has recently been developed [2]–[5]. The goal of lung cancer immunotherapy is to augment the weakened host immune response against tumors using specific and/or nonspecific immune stimulants [4]–[6]. Nonspecific immunostimulatory agents and interventions with cytokines have limited clinical benefits. The target-directed immunotherapy with defined tumor antigens, such as melanoma-associated antigen 3 and mucin 1(MUC1), are suboptimal and strong adjuvant agents are needed [6], [7]. In addition, it is now clear that lung cancer often present a tolerogenic microenvironment that hampers effective antitumor immunity. Therefore, new potent and efficacious immunotherapy, both augmenting antitumor immunity and counteracting tumor-mediated immunosuppression for lung cancer are needed.

Malaria, which is caused by an intracellular parasite from the *Plasmodium* genus, is the most common parasitic infection in humans. Human malaria parasite infection can produce periodic high fevers in the acute phase. Hyperthermia has been clinically used for the treatment of certain cancers [8]–[11]. Furthermore, malaria has been reported to stimulate host immune responses, such as promoting IFN-γ production, activating natural killer (NK) cells, γδ T cells and NKT cells, inducing the maturation of dendritic cells (DCs), and stimulating T-cell proliferation [12], [13], which are believed to be efficacious for combating some clinical cancers [14]–[16].

In this study, we propose that malaria parasite infection is therapeutic for lung cancer. The antitumor effect and immunological mechanisms of malaria infection was studied in a murine lung cancer model. We found that *Plasmodium yoelii* 17XNL (*P. yoelii* 17XNL) infection significantly inhibited the growth and metastasis of Lewis lung cancer (LLC). Furthermore, we demonstrated that malaria infection provided antitumor effect by inducing potent innate and adaptive antitumor immunity.

## Results

### Suppression of LLC growth and metastasis development in mice by malaria parasite infection

**Figure 1:**
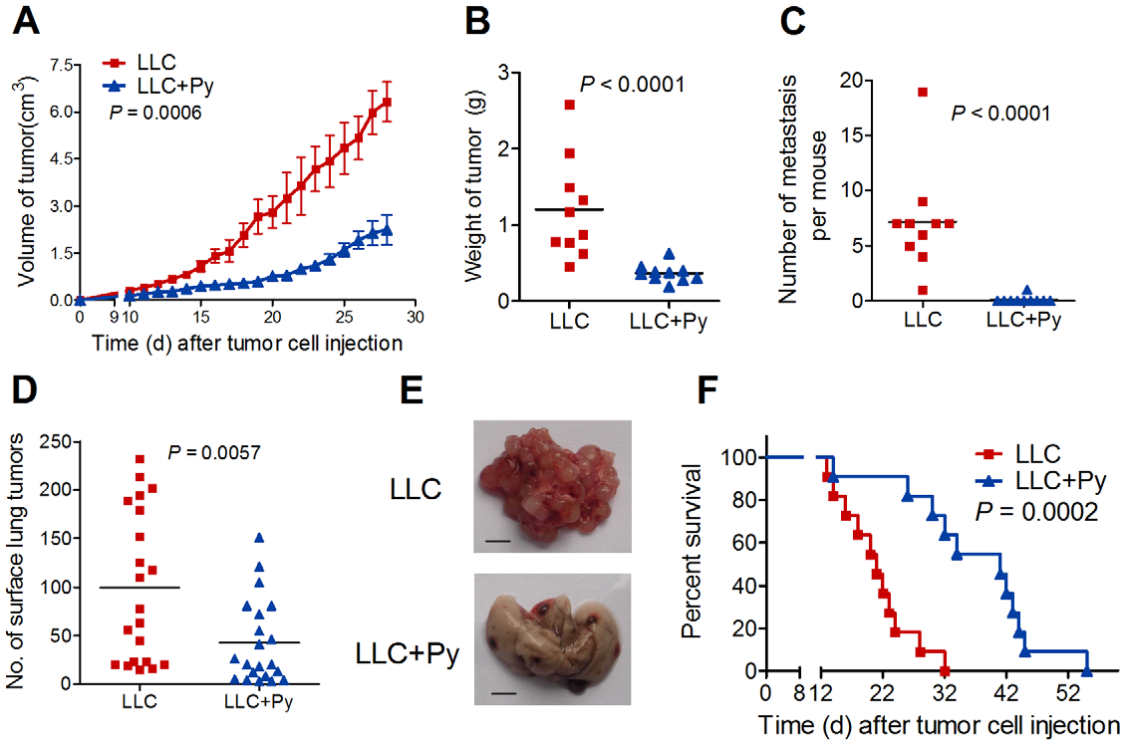
Malaria parasite infection suppresses tumor growth and metastasis in a mouse model. **A**, Tumor growth was measured over time (n = 12). The graph shows average with SD. **B**, Tumor mass 17 days after tumor cell inoculation (n = 10). **C**, Metastasis development 35 days after tumor inoculation. The graph shows the number of lung and liver tumors in the LLC and LLC+Py mice (n = 10). **D**, Number of lung tumor nodules in mice 18 days after intravenous tumor inoculation (n = 21). **E**, Examples of metastatic nodules in the lungs of mice in D. **F**, Survival curve of LLC and LLC+Py group mice (n = 11). All experiments were performed three times with similar results. Statistical differences between groups are indicated by the *P* values.

**Figure 2:**
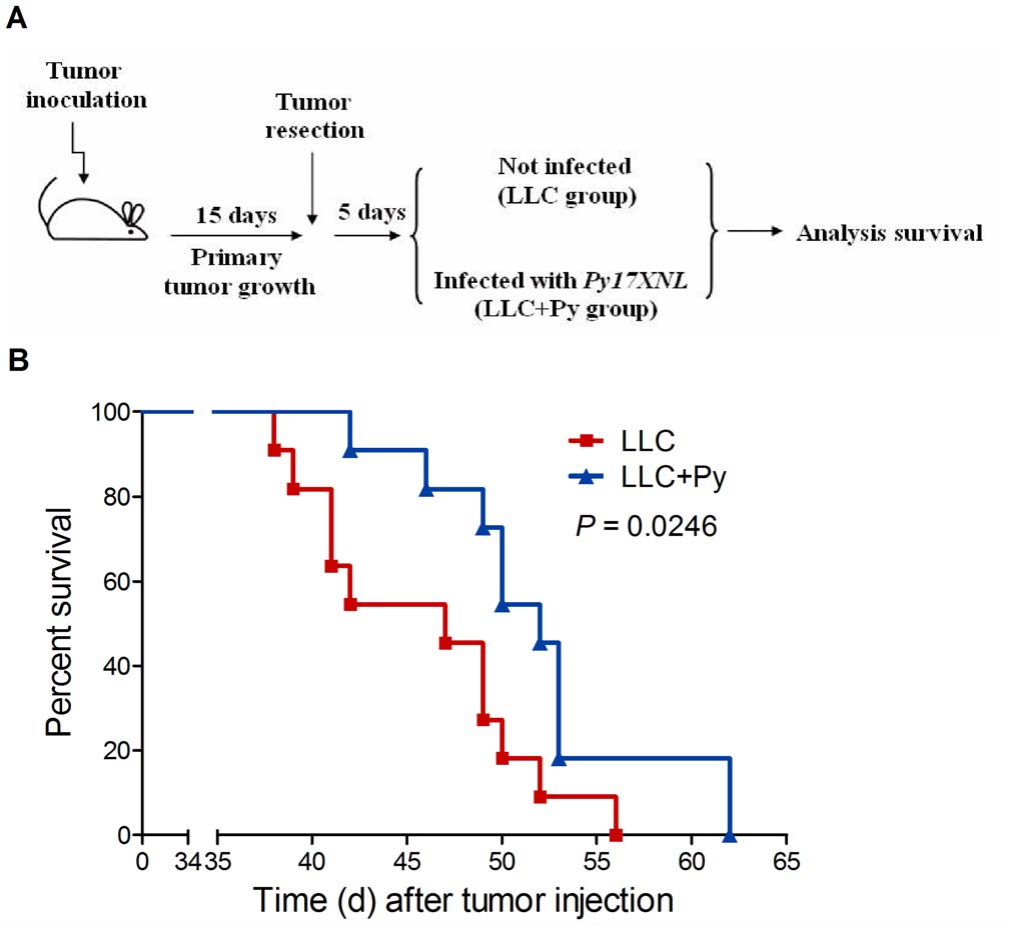
The antimetastatic effect of malaria infection after tumor surgery in mice. **A**, 5×10^5^ LLC cells were injected s.c. into the right flank of C57BL/6 mice. After 15 days of tumor growth, primary tumors were surgically removed. Mice were followed for an additional 5 days to recovery, then half of the mice were infected with *P. yoelii* 17XNL and the others were injected with an equivalent number of uninfected erythrocytes. **B**, Data shows the survival curves of mice after primary tumor removal. *P. yoelii* 17XNL infected mice survived much longer than their uninfected counterparts (n = 11).

To determine the effect of malaria infection on the growth of LLC cells, we infected tumor-bearing mice seeded with a subcutaneous (s.c.) injection of LLC cells with *P. yoelii* 17XNL parasitized erythrocytes (LLC+Py) or with an equivalent number of uninfected erythrocytes (LLC). During the infectious period of malaria (Fig. S1), the growth of tumor cells was clearly suppressed in the LLC+Py group compared to the LLC group (Fig. 1A&B). The tumor volumes (*P* = 0.0006, Fig. 1A) and tumor weights (*P*<0.0001, Fig. 1B) were significantly decreased in the LLC+Py group mice as compared with the LLC group mice. We then determined the effect of malaria parasite infection on lung cancer metastasis by analyzing the occurrence of distant metastases in the lung and liver on day 35 post-tumor cell inoculation. We found that whereas the LLC control group exhibited a high frequency of metastasis (10 of 10 mice, 100%), metastasis in the LLC+Py group was only observed in 1 of 10 animals (*P*<0.0001, Fig. 1C and Fig. S2). This experiment was then performed using a more aggressive metastatic model produced by intravenously (i.v.) injection of tumor cells into the mice [17]. Similar to the previous s.c. tumor model, malaria parasite infection significantly reduced the number of metastases that developed in the lung (*P* = 0.0057, Fig. 1D&E). In addition, malaria parasite-infected tumor-bearing mice survived much longer than their uninfected counterparts (*P* = 0.0002, Fig. 1F). Distant metastases are a major reason for cancer-related death in patients with solid tumors [18]. Therefore, we combined surgical treatment and malaria infection to verify its antimetastatic activities. The primary tumor was surgically removed after tumor formation. Five days later, half of the mice were infected with *P. yoelii* and the others were uninfected (Fig. 2A). The results showed that parasite-infected tumor-bearing mice survived much longer than their uninfected counterparts after surgery (*P* = 0.0246, Fig. 2B). This observation hints toward effects of malaria parasite infection against late steps in the metastatic process after invasion of the blood vessels by metastatic tumor cells.

### Effect of malaria parasite infection on tumor cell proliferation, apoptosis, and angiogenesis

**Figure 3:**
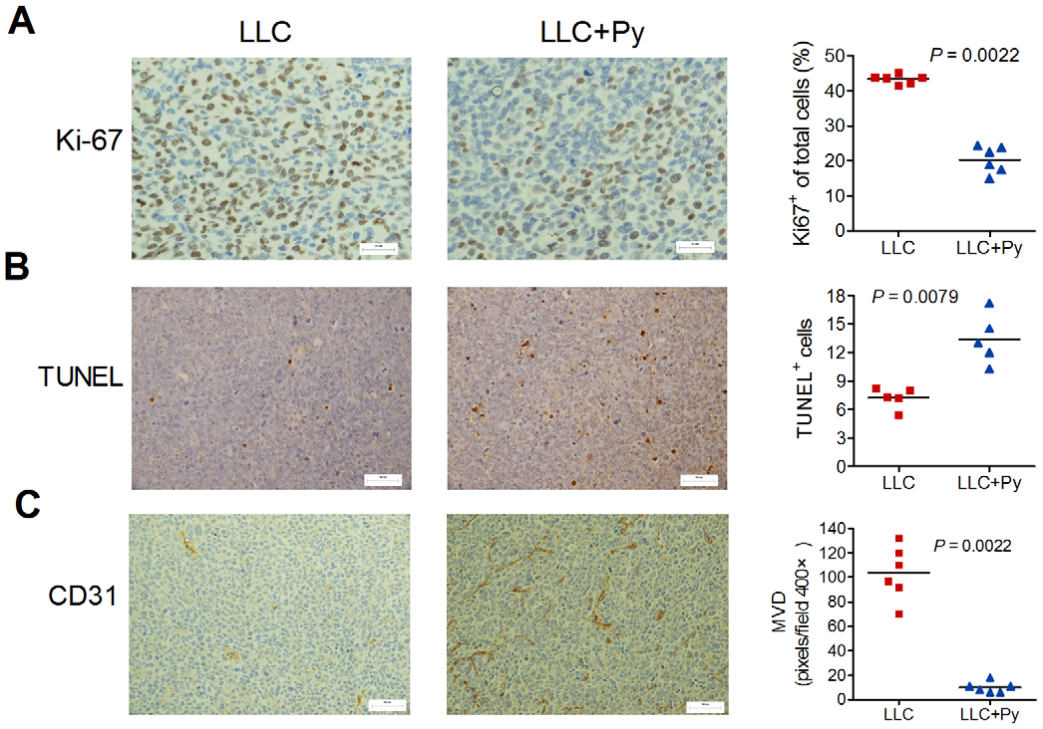
Effect of malaria parasite infection on tumor cell proliferation, apoptosis, and angiogenesis. **A**–**C**, Immunohistochemical staining (left) and assay quantification (right, n = 5–6) for Ki-67 (A), TUNEL (B), and CD31 (C) analyses of the tumor samples in Fig. 1B. Each symbol corresponds to an individual animal. The line represents the mean value. Bars: 25 µm in A, 50 µm in B and C.

As shown in Figure 3, staining for the proliferation marker Ki67 shows significant suppression of proliferation in the LLC+Py group mice, compared with the LLC group mice (*P* = 0.0022, Fig. 3A). Meanwhile, apoptosis cells were markedly increased within tumors of the LLC+Py group mice versus the LLC group mice (*P* = 0.0079, Fig. 3B), as evidenced by terminal deoxynucleotidyl transferase-mediated (TUNEL) staining of tumors at day 17. Then, tumor blood vessels were examined to determine whether the observed decrease in proliferation and increase in apoptosis were affected by vascular pruning [19]. Staining of endothelial cells in blood vessels with CD31 showed extensive tumor vasculature between tumor nests in the LLC group mice versus marked angiogenic suppression in the LLC+Py group mice (*P* = 0.0022, Fig. 3C and Fig. S3).

### Induction of Th1-type cytokines and increasing of NK-cell cytotoxicity activity and infiltration

**Figure 4:**
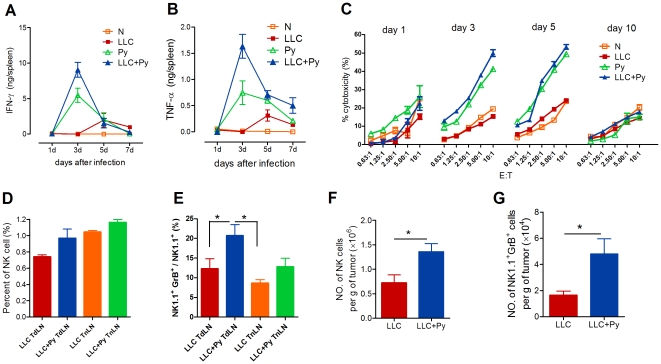
Malaria parasite infection induces the production of Th1-type cytokines and increases NK cell cytotoxicity activity. **A** and **B**, Levels of IFN-γ (A) and TNF-α (B) in splenocyte culture supernatants as measured by ELISA (n = 4). **C**, The cytotoxicity of NK cells enriched from splenocytes at various time points was assessed against YAC-1 cells at the indicated effector-to-target (E∶T) ratios (n = 4). **D** and **E**, Five days after tumor inoculation, the percentage (D) and the granzyme B-secretion (E) of NK cells were determined in TdLN and TnLN of mice by flow cytometry (n = 6). **F** and **G**, The absolute numbers of tumor-infiltrating NK cells (F) and spontaneous granzyme B-producing NK cells (G) were quantified by flow cytometry and normalized to biopsy weight (n = 6) 17 days after tumor inoculation. These experiments were independently performed three times with identical results, and the representative scatter plots are shown. All graphs show average with SD. *, *P*<0.05.

The potent anti-tumor activity of malaria infection greatly encouraged us to further explore its profound mechanism. The innate immune system which is believed to produce protective cytokines and activate innate lymphocytes provides the way to quickly resist tumor [20]. We found that malaria parasite infection led to rapid increases in IFN-γ and TNF-α levels, peaking 3 days after infection (Fig. 4A&B), and the lytic activity of NK cells was significantly increased during the early stages of infection (Fig. 4C). Although the percentage of NK cells in tumor-draining lymph nodes (TdLNs) was not significantly different between the groups (Fig. 4D), parasitic infection increased the number of granzyme B-secreting NK cells in TdLNs (Fig. 4E). An increase in both the number of NK cells and granzyme B-secreting NK cells was observed in tumor tissue in the LLC+Py group (Fig. 4F&G), suggesting that after parasitic infection, tumor-infiltrating NK cells exhibit higher *in situ* cytotoxic activity. These results confirmed that malaria infection had a function in the activation of the innate immune response that acts to control the growth of inoculated LLC cells.

### Induction of anti-tumor-specific immune responses

Because of the weak immunogenicity of LLC cells, studying the antigen (Ag)-specific immune response to LLC cells is challenging [21]. Therefore, we generated a recombinant LLC cell line (LLC-MUC1) that expressed MUC1 (Fig. S4) to examine the tumor Ag-specific immune responses induced by malaria parasite infection. In order to further confirm the immune responses to be tumor Ag-specific, not mouse-self Ag-specific, we used an exogenous MUC1 (human MUC1) to generate the LLC-MUC1 cell line. Human MUC1 is highly immunogenic in mice and its epitope is very clear so that it is convenient for us to examine the tumor Ag-specific immune responses. LLC-MUC1 cells and the parental LLC cells did not show any significant difference in growth and morphological characteristics in vitro, and also displayed a similar growth rate in vivo (data not shown).

**Figure 5:**
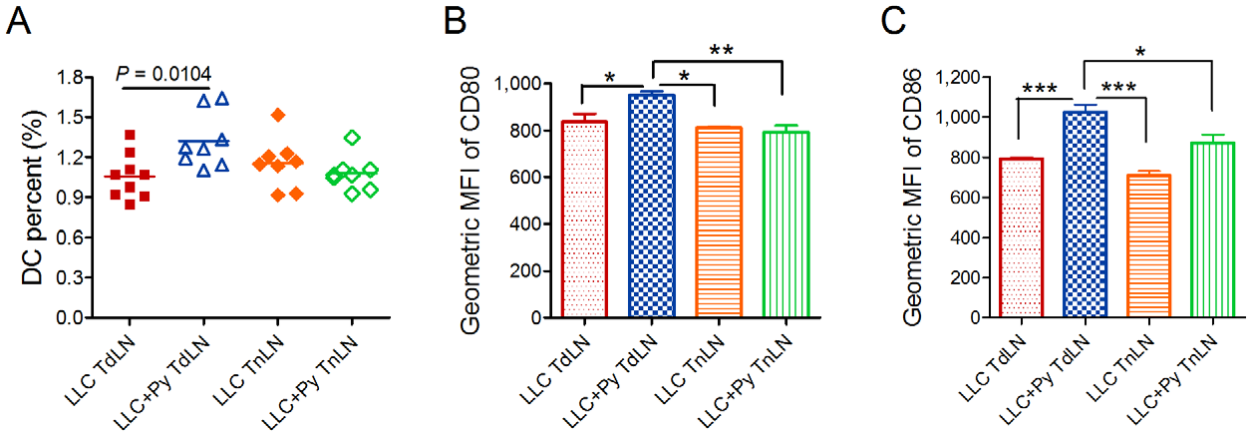
Malaria infection increases the percentage and maturation of DC in TdLN. Two days after tumor inoculation, DC maturation profile and numbers were determined in lymph node cells. **A**. Quantification of DC percentage in lymph nodes by flow cytometry. DC percentage in TdLNs and TnLN of tumor bearing mice are shown. The line in graph indicates the mean value. **B** and **C**, The geometric mean fluorescence intensity of CD80 (B) and CD86 (C) on DCs, data are pooled from three independent experiments. The graph shows average with SD. *P<0.05; ** *P*<0.01; *** *P*<0.001.

Given the central role of DCs in initiating and maintaining Ag-specific immune responses [20], [22]–[24], we analyzed the percentage and the maturation of CD11C^+^ DCs in the lymph node (LN) in tumor-bearing mice infected with *P. yoelii* 17XNL parasitized erythrocytes (LLC+Py) or uninfected erythrocytes (LLC). We found that malaria infection at early stage increased the percent of CD11C^+^ DCs (Fig. 5A and Fig. S5) and up-regulated CD80 and CD86 expression by CD11C^+^ DCs in the tumor draining lymph node (TdLN), as compared to the LLC group mice (Fig. 5B&C). This increase in the percentage and maturation of DCs was not observed in non-tumor draining lymph node (TnLN) of both groups of mice (Fig. 5 and Fig. S5).

**Figure 6:**
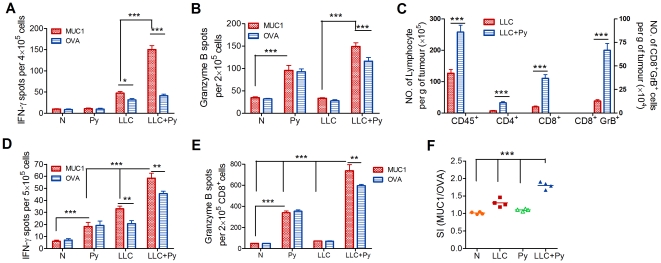
Malaria parasite infection induces anti-tumor-specific immune responses. **A** and **B**, IFN-γ- (A) and granzyme B- (B) producing cells derived from TdLNs 17 days after tumor inoculation were assessed by ELISPOT assay (n = 6). **C**, The absolute numbers of tumor-infiltrating CD45^+^ cells, CD4^+^ and CD8^+^ T cells, and spontaneous granzyme B-producing CD8^+^ T cells were quantified by flow cytometry and normalized to biopsy weight (n = 6) 17 days after tumor inoculation. **D** to **F**, Splenocytes were harvested 30 days after tumor inoculation. **D**, IFN-γ production was measured using an ELISPOT assay (n = 6). **E**, CD8^+^ T cells were enriched from splenocytes and subjected to ELISPOT assay to measure granzyme B production (n = 6). **F**, Proliferation of splenocytes was measured by BrdU incorporation (n = 6). Data are presented as the stimulation index (SI), which was calculated by determining the ratio of specific MUC1 peptides to non-specific OVA peptides. All graphs show average with SD. These experiments were independently performed three times with identical results, and the representative scatter plots are shown. *, *P*<0.05; **, *P*<0.01; ***, *P*<0.001.

We then assessed whether malaria infected-associated DCs enhancement translated into the improved cross-priming and intra-tumor recruitment of specific T cells or not [25], [26]. Using our tumor mouse model with LLC-MUC1 cells, we assessed the tumor-specific (MUC1 versus irrelevant OVA) production of IFN-γ and granzyme B in TdLN cells by enzyme-linked immunospot (ELISPOT) assay. We observed a significant increase in the numbers of IFN-γ/granzyme B-secreting effector cells in response to MUC1 (Ag-specific), but not to OVA, in the LLC+Py group (Fig. 6A&B). This suggests that TdLN DCs in LLC+Py group mice are competent to cross-prime peripheral tumor specific T cells, eliciting MUC1-specific immune responses with higher potency. To verify that the DC activated TdLN cells actually migrated into tumor tissue, the density of tumor-infiltrating leucocytes was examined. We found that in LLC+Py mice, there was a dramatic increase in the density of tumor-infiltrating CD45^+^ cells, and when tumor-infiltrating lymphocyte subsets were examined, both CD4^+^ and CD8^+^ T cells populations were increased with CD8^+^ T cells representing the dominant cell type (Fig. 6C). Among the CD8^+^ T cells, the density of granzyme B-expressing cells was also increased significantly in the tumors from the LLC+Py group (Fig. 6C). These data suggested that malaria infection promoted the accumulation of both CD4^+^ T cells and CD8^+^ T cells, and the CD8^+^ T cells had increased the effector function within the tumor.

We next examined the systemic anti-tumor immune response and observed that the number of MUC1-specific IFN-γ-producing cells in the spleen was much higher in the LLC+Py mice compared to the LLC mice (Fig. 6D). Among the granzyme B-producing cells in the spleen, the number of MUC1-specific CD8^+^ T cells was significantly higher in the LLC+Py group relative to the LLC group (Fig. 6E). We further measured tumor-specific T cell proliferation and found that the Ag-specific proliferative ability of splenocytes from the LLC+Py group mice was significantly higher than that of the LLC group mice (Fig. 6F). These data suggested that malaria infection induced the systemic anti-tumor immune response.

### Stimulation of effective, long-lasting anti-tumor immunity

**Figure 7:**
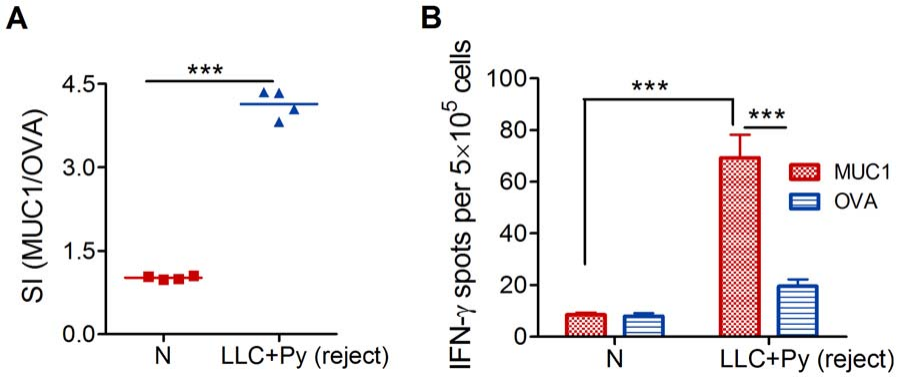
Malaria parasite infection stimulates effective long-lasting anti-tumor immunity. **A** and **B**, *P. yoelii* 17XNL infection resulted in complete tumor regression in 5–10% (1–2 per 20 mice) of the infected tumor-bearing mice. Fifty days after infection, splenocytes from the mice exhibiting tumor regression were analyzed for MUC1-specific proliferation (A) and IFN-γ secretion (B) (n = 4). The line in A indicates the mean value. Bars in B represent SD. ***P<0.001.

Malaria parasite infection resulted in prolonged survival and complete tumor regression in 5–10% (1–2 per 20 mice) of the infected tumor-bearing mice. The surviving animals rejected subsequent subcutaneous injections of parent tumor cells on day 50 after the initial tumor inoculation, suggesting that they had acquired systemic immunity against LLC cells. To assess whether malaria parasite infection led to the generation of long-term immunity, 50 days after tumor inoculation, splenocytes from the mice exhibiting tumor regression were analyzed for Ag-specific proliferation and IFN-γ secretion. Splenocytes from the mice exhibiting tumor regression proliferated considerably in response to MUC1 peptides (Fig. 7A) and produced high levels of Ag-specific IFN-γ (Fig. 7B), indicating that malaria parasite infection augmented not only *in situ* immunity but also long-lasting, systemic tumor-specific immunity, contributing to the inhibition of tumorigenesis and the rejection of tumor infiltration at distant sites.

### Antitumor effect of the combination of malaria parasite infection and DNA vaccine treatment

**Figure 8:**
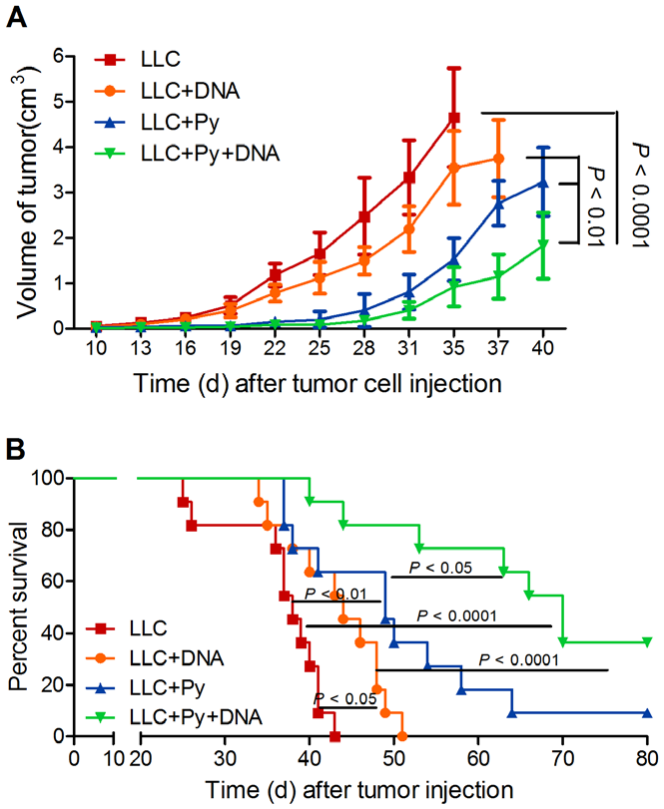
Combination of malaria parasite infection and DNA vaccine treatment produces a synergy antitumor effect. **A** and **B**, 5×10^5^ LLC-MUC1 cells were injected s.c. into the flank region of mice. Four groups (LLC, LLC+DNA, LLC+Py, and LLC+Py+DNA) of mice were immunized with DNA vaccine or infected with parasites as described in Materials and Methods and subsequently monitored for tumor development for 40 days (A) and determined the survival end point for 80 days (B). The graph of (A) shows average with SD. Statistical differences between groups are indicated by the *P* values.

Successful adjuvants help protective antigens to stimulate potent and long-lasting immune responses. In the case of malaria, there are several candidate molecules in malaria parasites that could act as adjuvant components [27], [28]. To evaluate whether the parasite infection could enhance the immune response of lung cancer DNA vaccine pcDNA3.1-hMUC1 [29], tumor-bearing mice were immunized with monotherapy (LLC+DNA) or combination therapy (LLC+Py+DNA), and animals infected with parasitized erythrocytes (LLC+Py) or uninfected erythrocytes (LLC) served as control groups. The results demonstrated that the combination of malaria parasite infection and DNA vaccine treatment exerted synergistic effects on the inhibition of tumor growth (Fig. 8A) and the prolongation of mouse survival (Fig. 8B). These data suggest that malaria parasite is a novel adjuvant that can facilitate specific antitumor immunity.

## Discussion

Tumors frequently interfere with the development and function of immune response [30]. The ability of various infections to suppress cancer growth has been well documented [31]–[34]. In the present study, we examined antitumor and antimetastatic activities of malaria infection in both subcutaneously and intravenously implanted murine LLC model. Malaria parasite infection inhibited LLC growth and metastasis and prolonged the survival of tumor-bearing mice. Further histological analysis of tumors from mice infected with malaria revealed that angiogenesis was inhibited and the proportion of proliferative cells was decreased, whereas apoptotic cells were increased. Malaria infection in LLC-bearing mice also significantly increased the secretion of IFN-γ and TNF-α, the activation of NK cells, tumor-specific T-cell proliferation, and cytolytic activity of CD8^+^ T cells. These results indicate that malaria parasite infection induces innate and adaptive antitumor responses in LLC-bearing mice, leading to the induction of antitumor and antimetastatic activities.

Lung cancer is the major cause of cancer-associated deaths. Its high fatality means that no effective treatment is available [1]. Immunotherapy may represent one of new therapeutic strategies for lung cancer has recently been developed. The goal of lung cancer immunotherapy is to augment the weakened host immune response against tumors using specific and/or nonspecific immune stimulants. Traditional biological immunoadjuvants, such as Bacillus Calmette-Guérin (BCG) and diphtheria toxin, and nonspecific immunostimulatory interventions with cytokines, such as IL-2, IFN-α, and TNF-α, induce a massive influx of inflammatory cells and the production of a Th1 cytokine profile that leads to affirmative efficacy in the treatment of some cancers [5]. However, the induction of the desired immune responses is weak, responses are short lived, and memory formation is defective. Thus, non-specific immune simulation has not shown a clear benefit for lung cancer in either preclinical or clinical efforts. Like most pathogens [31]–[34], malaria parasite induces a massive influx of inflammatory cells and production of a Th1 cytokine profile that leads to an immune response against tumor cells. Notably, malaria parasite infection induces not only non-specific antitumor immunity but also tumor-specific immunity. In addition, malaria infection generates effective and long-lasting local and systemic antitumor immunity.

Current antitumor specific immunotherapy such as therapeutic vaccines is still suboptimal. The immunoadjuvant component is needed to create a proinflammatory milieu, thereby enhancing costimulation and T lymphocyte activation [35]. In addition, the tumor microenvironment is potently immunosuppressive as a result of rapid functional inactivation of specific immunotherapy-induced effector lymphocytes that manage to home to the tumor microenvironment [6]. However, malaria parasite infection increases the percentage of DCs, up-regulates CD80 and CD86 expression of DCs in TdLN, promotes the maturation of DCs, and delivers costimulation signals that result in the generation of T cells with an activated phenotype. Activated CD4^+^ T cells produce an array of cytokines leading to the generation and clonal expansion of tumor-specific CD8^+^ T cells with cytolytic activity which recognize and kill tumor cells [20]. Furthermore, malaria parasite infection augments CD8^+^ T-cell-mediated antitumor immunity through increased number and effector function both in the TdLNs as well as in the tumor itself. Malaria parasite infection also promotes tumor-infiltrating NK cells that exhibit high cytotoxic activity in the tumor microenvironment.

Malaria parasite infections profoundly provoke the host immune system, inducing polyclonal activation, massive proliferation and differentiation of lymphocytes with parasite-unrelated specificities as well as immune responses to host antigens released during malaria parasite infection [36]–[41], which may serve as enhanced immune surveillance mechanisms against lung cancer. Possibly, the pre-existing antibodies and pre-activated non-tumor-specific responses together with the tumor-Ag-specific humoral and cellular responses initiated after the parasite infection might inhibit early stage tumorigenesis. Then, when malaria parasite infection is acquired during lung cancer growth and development, the innate and adaptive immune responses may be further induced and/or enhanced and strongly suppress tumor growth and metastasis. In addition, there are several candidate molecules in malaria parasites that could act as adjuvant components [27], [28]. The glycosylphosphatidylinositol (GPI) of *P. falciparum* is believed to be an important factor in the induction of proinflammatory responses mainly through Toll-like receptor(TLR)2/MyD88-mediated signaling [42]. Hemozoin, a bio-crystalline substance, is a hemin detoxification by-product of malaria parasites, have been shown to mediate innate immune system activation through TLR9 [27], [28]. Hemozoin also shows a potent adjuvant effect with several model antigens. The “built-in” adjuvant may facilitate the specific antitumor effect in our study. Taken together, the malaria parasite may be an ideal vaccine vehicle not only for presentation of lung cancer antigens (such as MUC1) to tumor-specific T-cells, but also act as an adjuvant to provide the appropriate milieu to enhance the efficacy of these effector cells [43]–[45].

However, our studies have two clear limitations. First, there is no fever in rodent malaria [46], [47], which was also observed in our study (data not shown). Therefore, there should be no hyperthermia effect in our rodent malaria-cancer models. Second, the anti-cancer effects have been confirmed to be much stronger in mice with relatively longer natural disease course (approximately four weeks) of *P. yoelii* infection compared to those with a shorter disease course (approximately two weeks) of *P. chabaudi* infection or those with interrupted short courses of *P. yoelii* infection (two weeks, following antimalarial drug treatment, data not shown). That means the longer infectious period it is, the stronger effect on the inhibition of lung cancer growth it will be. Unfortunately, the longest period of rodent malaria infection is only maintained four weeks (*P. yoelii*). So we couldn't observe the effect of long-term malaria infection on the cancer growth. However, one of the benign forms of human malaria parasite *P. vivax* produces periodic high fevers in the acute phase, and this parasite subsequently survives for years during the chronic phase of infection if the patient forgoes treatment [48], [49]. Hence, we hypothesize that a long course of therapeutic malaria for human lung cancer should be effective.

Despite Greentree proposed the macrophage activation-based hypothesis of therapeutic malaria (malariotherapy or malariatherapy) for cancer in 1981[50], the positive relationship between malaria and some virus-associated cancers (such as Burkitt's lymphoma and cervical cancer) has been well documented [51], [52]. However, we report here that malaria infection significantly suppresses LLC growth via induction of innate and adaptive antitumor responses, which provides a novel understanding of the relationship between malaria and lung cancer. We suggest that the urgent need is to explore the feasibility of using a gene-modified attenuated Plasmodium as a lung cancer vaccine vector and to identify the active cellular components of malaria parasite for immunotherapy for lung cancer.

## Materials and Methods

### Ethics Statement

The animal experiment facilities were approved by the Guangdong Provincial Department of Science and Technology, the approval ID is SYXK (Guangdong) 2005-0063, and complied with the guidelines of the Animal Care Committee, Guangzhou Institutes of Biomedicine and Health, Chinese Academy of Sciences (Animal Welfare Assurance #A5748-01). All surgery was performed under anesthesia, and all efforts were made to minimize animal suffering.

### Mice, cells, and parasites

Female 8- to 10-week-old C57BL/6 mice were purchased from the Vital River Experiment Animal Limited Company (Beijing, China) and raised in the Animal Center of the Guangzhou Institutes of Biomedicine and Health in accordance with the Guide for the Care and Use of Laboratory Animals established by this institute. The murine LLC cell line and YAC-1 lymphoma cells were obtained from the Chinese Academy of Sciences Cell Bank (Shanghai, China). The pcDNA3.1 plasmid expressing human mucin 1(MUC1) [29], obtained from Dr. June-Key Chung (Seoul National University College of Medicine, Seoul, Korea), was stably transfected into LLC cells with Lipofectamine (Invitrogen, Carlsbad, CA) [53] and selected with neomycin (G418; Sigma, Saint Louis, MO, USA) to generate an LLC-MUC1 cell line. MUC1 expression levels were confirmed by western blotting [53], [54]. The nonlethal *P. yoelii* 17XNL strain was obtained from the Malaria Research and Reference Reagent Resource Center (MR4).

### Tumor Growth and Metastasis Inhibition Assay

To establish tumors, C57BL/6 mice were s.c. injected with 5×10^5^ murine LLC cells. At the same time, the tumor cell-inoculated mice were infected with 5×10^5^ *P. yoelii* 17XNL-parasitized erythrocytes (LLC+Py). Mice injected with LLC cells and challenged with non-infected erythrocytes were used as the control group (LLC). Animals were examined daily until the tumors became palpable, after which the tumor volume was determined daily by measuring the diameter of the tumors using calipers. The volume was calculated using the formula, *V* = (*ab^2^*)/2, where *a* is the long axis, and *b* is the short axis. To assess tumor metastasis following primary tumorigenesis, 5×10^5^ LLC cells were injected s.c. into the right flank. After 35 days of tumor growth, the mice were sacrificed, and metastasis to the lung and liver was determined by counting individual metastatic nodules. Paraffin slides of tissue were analyzed for tumor cell apoptosis and proliferation, as well as tumor angiogenesis and metastasis as described previously [19]. To analyze the effect of malaria parasite infection on metastasis in the intravenous tumor cell model, the mice were injected with 5×10^5^ LLC cells i.v. via the tail vein and followed for 18 days before metastasis was analyzed. Metastasis development was evaluated by counting individual metastatic nodules. To evaluate the antimetastatic effect of malaria infection after surgery, 5×10^5^ LLC cells were injected s.c. into the right flank of C57BL/6 mice. After 15 days of tumor growth, primary tumors were surgically removed. Mice were followed for an additional 5 days to recover, and then half of the mice were infected with *P. yoelii* 17XNL and the others were injected with equivalent number of uninfected erythrocytes. The survival end point was determined by either spontaneous death of the animal or because of the presence of moribund signs. In all experiments, treatment groups were randomized to prevent cage effects.

### Lymphocyte isolation

The generation of single-cell suspensions from the spleen and lymph nodes was described previously [55], [56]. Isolation of NK and CD8^+^ T cells from splenocytes was performed using anti-mouse DX5 and CD8a MicroBeads, respectively, according to the manufacturer's instructions (Miltenyi Biotec, Bergisch Gladbach, Germany). The purity of the selected cell populations was determined by flow cytometry. For analysis of tumor-infiltrating lymphocytes, tumor-bearing mice were sacrificed 17 days after malaria parasite infection, and the subcutaneous tumors were harvested. To prepare single-cell suspensions, tumors were digested in a mixture of 0.1% collagenase type IV (Worthington, Lakewood, NJ, USA) and 0.01% DNase I (Roche, Indianapolis, IN, USA) in RPMI-1640. The tumors were then disrupted using a cell strainer, and viable lymphocytes were collected after carrying out cell separation in a Ficoll gradient [57].

### Flow cytometry

The following antibodies and their corresponding isotype controls (all purchased from eBioscience, San Diego, CA, USA) were used for staining: CD45-PE, NK1.1-APC, CD4-FITC, CD11c-FITC, CD80-PE, CD86-PE, CD3-Percp-cy5.5, CD3-PE, CD8-APC, and CD8-PE. After adding the appropriate antibody, the cells were incubated in the dark at 4°C for 30 minutes and then washed twice with FACS buffer (0.1% BSA and 0.05% sodium azide in PBS). For intracellular granzyme B staining, the cells were first stained with either CD8- or NK1.1-APC followed by fixation and permeabilization, and the cells were then stained with granzyme B-PE (eBioscience). Flow cytometry samples were acquired on a BD FACSAria and analyzed with FlowJo software (Tree Star, Inc.)

### Cytokine assays (ELISA)

Splenocytes were cultured at a concentration of 10^7^ cells/ml in RPMI-1640. After incubation for 48 h, culture supernatants were harvested and assayed for cytokine levels by two-site sandwich enzyme-linked immunosorbent assay (ELISA) [58].IFN-γ and TNF-α were assayed using pairs of capture and detection antibodies (R&D Systems, Minneapolis, MN, USA) according to the manufacturer's instructions. Cytokine levels were calculated using standard curves determined by recombinant murine cytokines.

### NK cell cytotoxicity

CytoTox 96nonradioactive cytotoxicity assay (Promega) was used to measure the specific lytic activity of NK cells in treated mice (4 mice per group) according to the manufacturer's protocol.

### Cell proliferation analysis

Splenocyte proliferation was assessed using a BrdU colorimetric ELISA (Roche) as recommended by the manufacturer. In brief, splenocytes were incubated with 10 µg/ml MUC1 (HGVTSAPDTRPAPGSTAPPA) peptides or the irrelevant peptide OVA (SIINFEKL) as a control for 72 h. 1×BrdU was added to the wells for 18 h and BrdU incorporation into newly synthesized DNA strands was detected exactly as recommended by the manufacturer.

### ELISPOT assay

All ELISPOT reagents were purchased from R&D Systems. Lymph node and spleen cells were used as responders in an IFN-γ and granzyme B ELISPOT assay as previously described [59]. Cells were stimulated with the MUC1 peptide (10 µg/ml) or OVA peptide (10 µg/ml, negative control). Briefly, PVDF membrane plates (Millipore, Bedford, MA, USA) were coated with an anti-murine capture antibody (R&D Systems) overnight at 4°C. Effector cells were added to triplicate wells at specified concentrations, then co-cultured with the MUC1 peptide (10 µg/ml) or OVA peptide (10 µg/ml, negative control) for 18 h at 37°C. The plates were washed and the biotinylated anti-murine detection antibody was added. Plates were incubated for 2 h, and a Streptavidin-conjugated alkaline phosphatase (1∶100 in PBS-T, R&D Systems) was added for 1 h. Spots were visualized with BCIP/NBT phosphatase substrate (R&D Systems) and subjected to automated evaluation using the ELISPOT Reader (CTL Limited). The mean numbers of spot-forming cells were calculated from the triplicate assays.

### Therapeutic Combination

To evaluate the combination effect of malaria parasite infection and DNA vaccine (expressing MUC-1) treatment, 5×10^5^ LLC-MUC1 cells (recombinant cell line that expresses MUC1, Fig S4) were injected s.c. into the flank region. The mice were then divided randomly into 4 groups (n = 11): LLC, LLC+DNA, LLC+Py, and LLC+Py+DNA. The parasite infection groups were injected i.p. with 5×10^5^ *P. yoelii* 17XNL-parasitized erythrocytes on the same day. The control mice received an equivalent number of uninfected erythrocytes. The DNA vaccine-treated groups were immunized intramuscularly on day 4, 10 and 15 after tumor inoculation. The development of tumors in individual mice was monitored every 3 days. The survival end point was determined by either spontaneous death of the animal or because of the presence of moribund signs.

### Statistical Analyses

For parametric data, differences between groups were analyzed with unpaired two-tailed Student's *t*-tests; for nonparametric data, differences between groups were analyzed with the Mann-Whitney test. Survival curves were analyzed by a log-rank test. All statistical analyses were performed with GraphPad Prism software.

## Supporting Information

Figure S1 **Dynamics of parasitemia in mice.** Parasitemia (percentage of infected erythrocytes versus total red blood cell count) in naïve C57BL/6 mice (Py) and LLC-bearing C57BL/6 mice (LLC+Py) infected with *Plasmodium yoelii* 17XNL. Blood smears were performed every 3 days, stained with Giemsa, and counted, n = 12. The graph shows average with SD.
(TIF)

Figure S2 **Malaria parasite infection prevents metastasis formation in mice.** H&E-stained paraffin slides of the lungs and livers of tumor-bearing mice infected with *P. yoelii* 17XNL (LLC+Py) or that were infected with an equivalent number of uninfected erythrocytes (LLC). Arrows show metastasis of tumors in the lungs and livers of mice.
(TIF)

Figure S3 **Malaria parasite infection inhibits tumor angiogenesis.** H&E-stained paraffin slides of tumor tissues from tumor-bearing mice infected with *P. yoelii* 17XNL (LLC+Py) or that were infected with an equivalent number of uninfected erythrocytes (LLC). Arrows show blood vessels within the tumor.
(TIF)

Figure S4 **Expression of MUC1 in recombinant LLC cells (LLC-MUC1).** The lysates of LLC-MUC1 or LLC cells were separated by SDS-PAGE, blotted, and probed with anti-MUC1 antibody and anti-GAPDH antibody as controls.
(TIF)

Figure S5 **Malaria infection increases the percentage of DC in TdLN.** Four days after tumor inoculation, quantification of DC percentage in lymph nodes of mice by flow cytometry. DC percentage in TdLN and TnLN of tumor bearing mice are shown. The graph shows average with SD. *** *P*<0.001.
(TIF)
