# Supplementary figures and images for: Antitumor Effect of Malaria Parasite Infection in a Murine Lewis Lung Cancer Model through Induction of Innate and Adaptive Immunity
Source: PLoS One. 2011 Sep 9;6(9):e24407. doi: 10.1371/journal.pone.0024407 (PMC3170332; doi:10.1371/journal.pone.0024407)

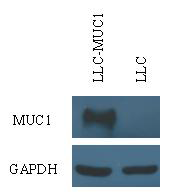

Supplement: Figure S4 — Expression of MUC1 in recombinant LLC cells (LLC-MUC1). The lysates of LLC-MUC1 or LLC cells were separated by SDS-PAGE, blotted, and probed with anti-MUC1 antibody and anti-GAPDH antibody as controls. (TIF) [file pone.0024407.s004.tif]

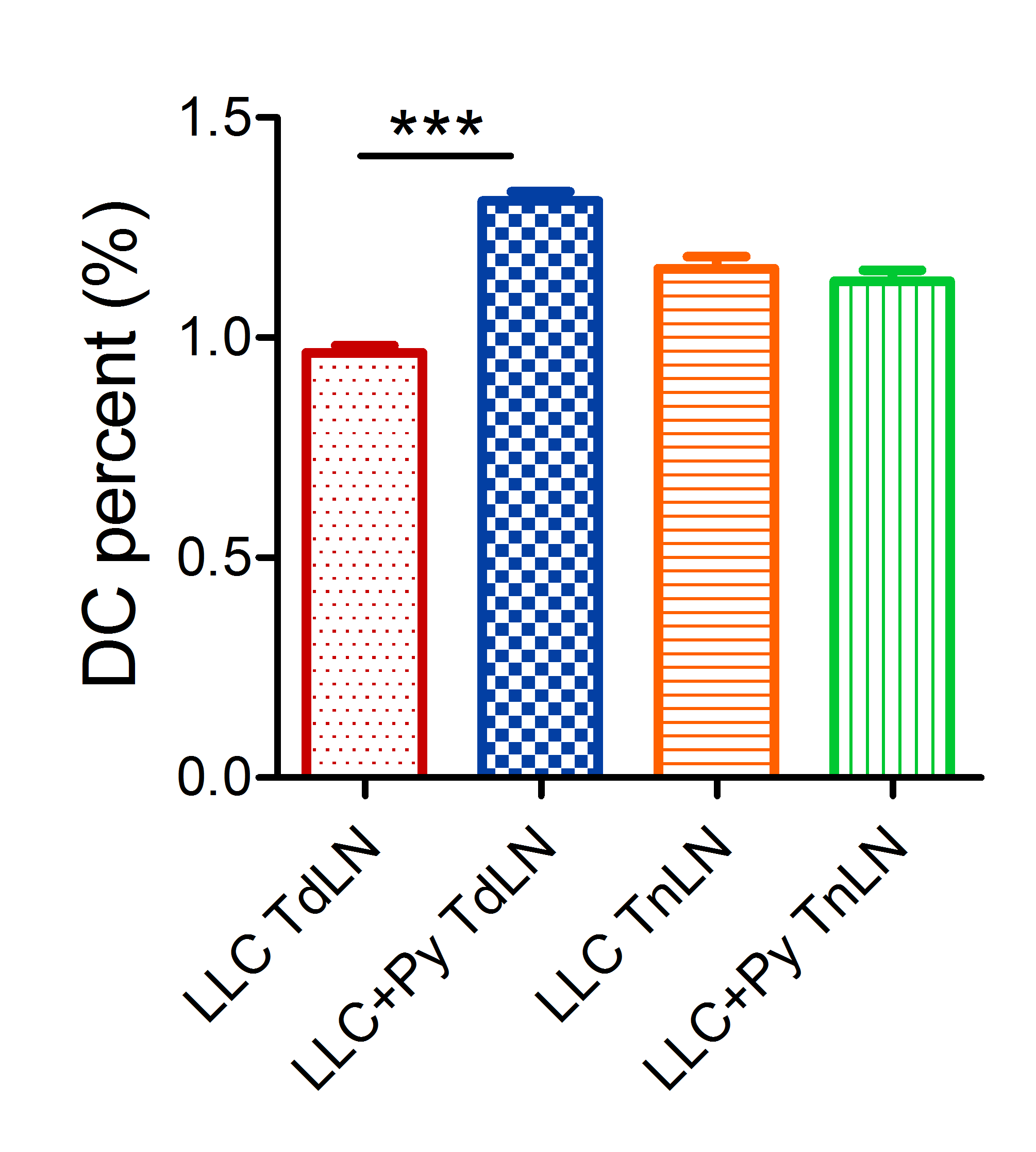

Supplement: Figure S5 — Malaria infection increases the percentage of DC in TdLN. Four days after tumor inoculation, quantification of DC percentage in lymph nodes of mice by flow cytometry. DC percentage in TdLN and TnLN of tumor bearing mice are shown. The graph shows average with SD. *** P<0.001. (TIF) [file pone.0024407.s005.tif]
